# Multi trace element profiling in pathogenic and non-pathogenic fungi

**DOI:** 10.1016/j.funbio.2020.03.001

**Published:** 2020-05

**Authors:** Silvia Wehmeier, Emma Morrison, Anthony Plato, Andrea Raab, Jörg Feldmann, Tina Bedekovic, Duncan Wilson, Alexandra C. Brand

**Affiliations:** a School of Medicine, Medical Sciences and Nutrition, University of Aberdeen, Foresterhill, Aberdeen, AB25 2ZD, UK; b TESLA, School of Natural and Computing Sciences, University of Aberdeen, Meston Walk, Aberdeen AB24 3UE, UK; c Medical Research Council Centre for Medical Mycology at the University of Exeter, Stocker Road, Exeter EX4 4QD, UK

**Keywords:** Calcium, *Candida albicans*, Fungal cell wall

## Abstract

Maintaining appropriate levels of trace elements during infection of a host is essential for microbial pathogenicity. Here we compared the uptake of 10 trace elements from 3 commonly-used laboratory media by 3 pathogens, *Candida albicans*, *Cryptococcus neoformans* and *Aspergillus fumigatus*, and a model yeast, *Saccharomyces cerevisiae*. The trace element composition of the yeasts, *C. albicans*, *C. neoformans* and *S. cerevisiae*, grown in rich (YPD) medium, differed primarily in P, S, Fe, Zn and Co. Speciation analysis of the intracellular fraction, which indicates the size of the organic ligands with which trace elements are complexed, showed that the ligands for S were similar in the three fungi but there were significant differences in binding partners for Fe and Zn between *C. neoformans* and *S.cerevisiae*. The profile for Cu varied across the 3 yeast species. In a comparison of *C. albicans* and *A. fumigatus* hyphae, the former showed higher Fe, Cu, Zn and Mn, while *A. fumigatus* contained higher P, S Ca and Mo. Washing *C. albicans* cells with the cell-impermeable chelator, EGTA, depleted 50–90 % of cellular Ca, suggesting that a large proportion of this cation is stored in the cell wall. Treatment with the cell wall stressor, Calcofluor White (CFW), alone had little effect on the elemental profile whilst combined Ca + CFW stress resulted in high cellular Cu and very high Ca. Together our data enhance our understanding of trace element uptake by pathogenic fungi and provide evidence for the cell wall as an important storage organelle for Ca.

## 1 Introduction

All organisms require major, minor and trace elements for growth. Phosphorus (P) is the most abundant as it is a key component of nucleic acids and membrane phospholipids. Sulphur (S) is the characteristic element found in the amino acids, cysteine and methionine. Magnesium (Mg) acts in many metabolic pathways and is important for genome stability, while calcium (Ca) is a second messenger in signalling transduction during growth and in response to external stimuli. Next in abundance are the transition metals, iron (Fe), zinc (Zn), copper (Cu), manganese (Mn), molybdenum (Mo) and cobalt (Co). Fe, Zn and Cu play structural roles in proteins and catalytic roles in enzymes, e.g., metallothionein and superoxide dismutase. A diverse array of metalloproteins require manganese for function, including oxidoreductases, DNA and RNA polymerases and protein kinases. Mo is an essential cofactor for enzymes catalyzing diverse key reactions in carbon, S and nitrogen metabolism. Co plays a crucial role in biological functions in the form of vitamin B12 and several metalloproteins. The importance of metal ions in biology is evidenced by the fact that 25 % of proteins in the Protein Data Bank coordinate a metal ion and almost half of all enzymes require a metal cofactor for function (Waldron et al., 2009). However, microbial metalloproteomes are largely uncharacterised (Cvetkovic et al., 2010). The metal content of cells can be accurately measured using inductively coupled plasma mass spectrometry (ICP-MS) and is enhanced by element speciation analysis, which identifies the chemical form (e.g oxidation state) of the element or size of the species (e.g. metalloprotein) to which the element is bound. This provides information about the mobility, bioavailability, impact and function of metals in biological systems (Ferrarello et al., 2002, Clemens, 2019).

Although essential for life, trace metals can also be highly problematic for the cell and require strict management. Some cations are toxic, such as Fe and Cu, which can generate reactive oxygen species that damage cellular components. Other mechanisms of toxicity depend on the ion’s position in the Irving–Williams series, which orders metals according to the stability of the complexes they form with organic ligands (Irving and Williams, 1953). Ions high on the scale, such as Cu and Zn, can displace the native cofactor of essential enzymes, rendering them non-functional (Shaw, 1961). In order to avoid metal toxicity, cells bind these cations (e.g. using metallothioneins) and store them in sub-cellular organelles (Perego and Howell, 1997, MacDiarmid et al., 2003, Zhao et al., 2020). Some ions, such as Ca, act as signalling molecules and their localized concentrations must be maintained through compartmentalization through a system of efflux pumps and antiporters. In filamentous fungi, a tip-high cytoplasmic Ca gradient is maintained, which confines growth to the hyphal tip (Schmid and Harold, 1988). In fungal pathogens, Ca is a second-messenger in the cell-integrity pathway, where it activates calcineurin-regulated responses (Blankenship and Heitman, 2005, Cruz et al., 2002, Reedy et al., 2010). Therefore, although cells contain millimolar amounts of Ca, local concentrations are held at the nM level so that very small changes can be detected.

The mechanisms evolved by fungi for the uptake of trace elements reflect their availability and the chemistry of the native environment. However, the scavenging mechanisms of opportunistic pathogens must also be able to function within the host environment, where strategies are in place to withhold trace elements from infectious microbes. Termed ‘nutritional immunity’, host restriction mechanisms include Fe sequestration in proteins, such as haem, and storage in ferritin (Weinberg, 1974), whilst extracellular iron is tightly chelated by transferrin and other molecules. Zn and Mn are withheld by binding to host S100A proteins such as calprotectin, psoriasin and calgranulin (Cunden and Nolan, 2018), although calprotectin has recently been shown to also chelate (ferrous) Fe and Cu (Besold et al., 2018, Nakashige et al., 2015).

Fungal disease kills approximately 1.5 million people per year and approximately half of these deaths are caused by 3 opportunistic pathogens – *Candida albicans*, *Cryptococcus neoformans* and *Aspergillus fumigatus* – which are the focus of research in medical mycology laboratories around the world (Brown et al., 2012). *C. albicans* is a human commensal of epithelial surfaces and the GI tract, but can become pathogenic in immunocompromised patients if introduced into the bloodstream. *C. neoformans* and *A.* *fumigatus* are environmental fungi that have the ability not only to grow at the high human body temperature of 37 °C (which is rare within the fungal kingdom) but have evolved mechanisms to scavenge trace metals within human tissue, an otherwise non-native environment. Studies of nutritional immunity suggest that the battle for trace elements within the host contributes to disease outcome (Mackie et al., 2016, Haley and Gaddy, 2016). For example, *C. albicans* scavenges extracellular Zn through secretion of a Zn-binding protein, Pra1, but this protein can also recruit host neutrophils to the site of infection (Citiulo et al., 2012, Luo et al., 2009, Soloviev et al., 2007, Soloviev et al., 2011). An understanding of the concentration of trace elements in the laboratory media employed in the study of nutritional immunity is therefore of value to the medical mycologist. The importance of Zn during infection is underscored by the fact that *A. fumigatus*, *Cryptococcus gattii*, *C. neoformans* and *C. albicans* mutants lacking Zn importers exhibit reduced virulence or *in vivo* fitness in models of invasive infection (Amich et al., 2014, Crawford et al., 2018, Schneider et al., 2015).

In this study, we first assessed the availability and uptake of trace metals by *C. albicans* in two fungal growth media. We next undertook quantitative studies of the total trace element content of cells in 3 comparative contexts: the yeast cells of the pathogens, *C. albicans* and *C. neoformans*, compared to the non-pathogenic model fungus, *Saccharomyces cerevisiae*; the hyphal and yeast growth morphologies of *C. albicans*; the hyphal form of *C. albicans* compared to the constitutively filamentous pathogen, *A. fumigatus*. For the yeasts, we also examined the cytoplasmic speciation profiles for 5 trace elements, including the transition metals, Fe, Cu, Zn and Mo. Lastly, we quantified the effect on the trace element profile of treating *C. albicans* cells with Calcofluor White and high extracellular Ca, a standard laboratory method for inducing cell wall stress in the study of antifungal drugs (Lee et al., 2012, Walker et al., 2008).

## 2 Materials and methods

### 2.1 Strains, media and culture conditions

Fungal strains used in this study are shown, along with their original source, in Table 1 (Fonzi and Irwin, 1993, Ralser et al., 2012, Smith et al., 1994). All glassware was washed in 10 % nitric acid to avoid background metal contamination. For analysis of fresh growth medium, 2 ml was retained from each batch prior to inoculation of cells. The same lots of all media components (yeast extract, peptone, etc.) were used throughout the study. For growth in YPD [1 % w/v yeast extract (Oxoid, UK), 2 % w/v mycological peptone (Oxoid), 2 % w/v glucose (Sigma, UK)], cells were inoculated to OD_600nm_ = 1 in 30 ml (*C. albicans* or *C. neoformans*) or by picking a single colony into 50 ml YPD (*S. cerevisiae*) and grown overnight at 30 °C with shaking at 200 rpm. For cell wall stress experiments, 30 ml YPD was supplemented with 0.2 M CaCl_2_ (Sigma), and/or 100 μg/ml Calcofluor White, as required, and/or washed twice with 0.02 M EGTA (ethyleneglycol bis-(β-aminoethylether)-N,N-tetraacetic acid). For speciation analysis, cells were grown in 120 ml YPD at 30 °C with shaking at 200 rpm. For the comparison of *C. albicans* yeast and hyphal biomass, cells were inoculated to OD = 0.05 in 200 ml MSM (Modified Soll’s Medium) Buffo et al. (1984), supplemented with biotin (0.001 g/L), arginine (0.0697 g/L) and trace metals (final concentrations: ZnSO_4_ 0.2 nM, FeCl_3_ 0.001 μM, CuSO_4_ 0.25 nM) (Sigma) at pH 4.5 for yeast and pH 6.8 for hyphae. Yeast was grown at 30 °C for 24 h and hyphae at 37 °C for 8 h. For growth in Complete Medium (Kaminskyj, 2001), *C. albicans* or *A. fumigatus* were inoculated into 30 ml medium and grown overnight at 37 °C. For biomass quantification and total element analysis, cell pellets were collected by centrifugation for 5 min at 4000 rpm, washed twice with milliQ water, dried in a drying oven at 70 °C overnight and the biomass (g) determined by subtraction from the tube weight.

**Table 1:**
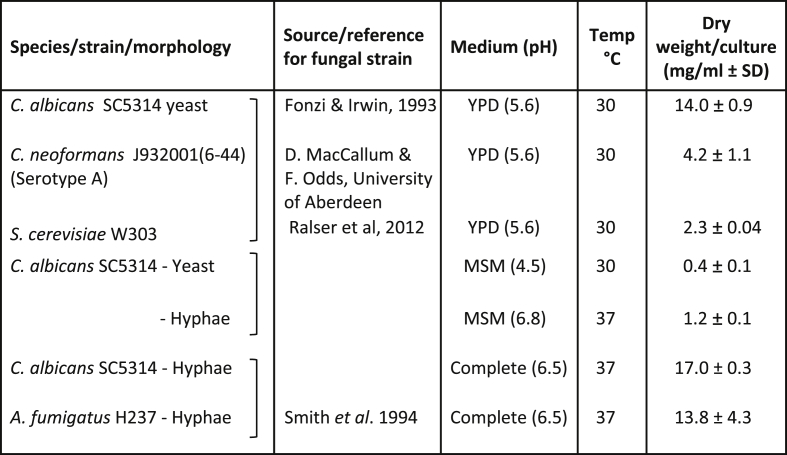
Biomass yields vary by species, morphology and medium.

The biomass yield (dried cell weight) of the yeast form of the pathogens, *C. albicans* and *C. neoformans,* were compared with that of the non-pathogenic obligate yeast, *S. cerevisiae*, by growing cells for 18 h in YPD pH 5.6 at 30 °C. Yields from the yeast and hyphal forms of *C. albicans* were obtained from growth in MSM pH 4.5 at 30 °C for 24 h (yeast) or pH 6.8 at 37 °C for 8 h (hyphae). Yields for *C. albicans* and *A. fumigatus* hyphal filaments were obtained by growing cells in Complete Medium pH 6.5 at 37 °C for 18 h. SD = standard deviation, n = 3.

### 2.2 Trace element quantification

For total element determination, dried cell pellets were digested overnight in PP-tubes (Corning, UK) using 1 ml conc. HNO_3_ (70 %, p.a. Sigma, UK) and 2 ml H_2_O_2_ (30 % p.a. Sigma, UK) in a high-performance microwave digestion unit, Mars5 (CEM, UK) using 5 min at 50 °C, 5 min at 75 °C and 30 min at 95 °C. After cooling, the samples were diluted to 10 ml in MilliQ water (Millipore, UK) and measured using an Element 2 ICP-MS (ThermoScientific, UK) in low, medium and high-resolution depending on isotope measured. Germanium and rhodium (each 10 μg/kg) were added via a T-piece before the nebulizer as online internal standard. For quantification standards, solutions were prepared from AccuTrace Standard mix, molybdenum, boron, antimony were added from standard stocks (1000 mg/L, BDH, UK). Reference materials (TORT-2 and DOLT-4 from NRC, Canada, RM 8415 from NIST, USA, BCR185R (IRMM, Belgium)) were used for quality control and treated like the samples (Raab et al., 2016).

For analysis of trace elements in the intracellular fraction, washed cell pellets were broken in 10 ml milliQ with acid-washed glass beads using a Fast-Prep at 10 cycles (6.5 m/s, 60 s bursts alternated with cooling). Cell lysates were centrifuged for 30 min at 10,000 rpm, concentrated through spin columns and stored at −20 °C. Cytosolic compounds were extracted using 50 mM TRIS/HCl buffer at pH 7.4 with a protease inhibitor and concentrations were standardised after quantification by Bradford assay. Samples (100 μl) were loaded into an Agilent 1100 HPLC system with a Superdex 75 (10∗300 mm) column (Pharmacia, UK) with a flow rate of 1 ml/min with the same buffer. The column was coupled directly with an Element 2 ICP-MS (inductively coupled plasma mass spectrometer) (ThermoScientific, UK) in medium resolution mode. Data were exported as csv-files and imported into Excel (Meharg et al., 2012).

### 2.3 Statistical analyses

Statistical analysis was undertaken in SPSS V26 using 2-tailed Student’s t-tests to compare individual elements pairwise with and without a treatment. Excel was used for calculation of correlation coefficients.

## 3 Results and discussion

### 3.1 Biomass yields vary by species and growth media

Interest in understanding host-pathogen trace metal interactions within the context of nutritional immunity has increased in recent years. However, the background levels of trace metals in laboratory media can confound data interpretation regarding the role of micronutrient uptake systems in pathogenicity. For example, the fungal zincophore, Pra1, is essential for endothelial cell damage only when exogenous zinc is absent from the surrounding culture media (Citiulo et al., 2012). The first aim of this study was therefore to assess the elemental profile of commonly-used laboratory culture media and, importantly, to quantify the levels of trace elements assimilated by the human fungal pathogens *C. albicans*, *C.* *neoformans* and *A. fumigatus*. The model yeast, *S. cerevisiae,* was used as a comparator.

We first assessed the biomass of each species following growth in the culture media that permitted the desired comparisons: YPD, for yeast growth of *C. albicans*, *C. neoformans* and *S. cerevisiae*; Modified Soll’s Media (MSM) for comparing yeast vs hyphal growth of *C. albicans*; and Complete media for comparing hyphal growth of *C. albicans* and *A. fumigatus*. The yield of dry weight biomass per ml of culture medium was determined for each fungus/morphology (Table 1). Of interest was the 3-fold higher biomass yield for *C. albicans* hyphae grown in Modified Soll’s Medium (a buffered, minimal amino acids/glucose medium) at near-neutral pH (6.8) at 37 °C compared to its yeast form grown in the same medium at acidic pH (4.5) and 30 °C. This may partly be due to the extensive vacuolation of sub-apical compartments in hyphae that allows tip growth to be maintained without the cost of generating the large volumes of new cytoplasm required for growth as yeast (Gow and Gooday, 1982).

### 3.2 Trace element availability in rich (YPD) or poor (MSM) medium

Studies of cell stress may be affected by the availability of trace elements in the growth medium, as trace metals are required for cellular stress responses (Crawford and Wilson, 2015, Eide, 2011). We therefore compared the availability of elements in newly-prepared YPD and MSM prior to inoculation with cells. YPD is an undefined rich medium and contained more of each element than the defined minimal medium, MSM, with the exception of S (Table 2). The composition of MSM gives Mg, Fe, Cu and Zn at trace levels with no added Ca, yet Ca was found to be present at 12 μM. Background levels of Ca from laboratory equipment, even when glassware is acid-washed, has been previously estimated to be ∼5 μM (Buffo et al., 1984). One of the largest differences between the media was for Mg, which was measured at 804 μM in YPD compared to 9 μM in MSM. To compare the assimilation of elements by *C. albicans* yeast in these two media, cells were grown to stationary phase and the cell pellets dried. Elements were quantified by weight and subtracted from the values obtained from fresh media to determine the percentage of each element that had been taken up by the fungus during growth. Mg and Zn were taken up at high levels from both media yet ∼25–30 % of these elements remained in the medium. This is consistent with the role of these elements as co-factors for a large number of proteins (Andreini et al., 2009). Uptake of Ca was relatively low in both media, perhaps reflecting the role of this element in signalling rather than structural functions. The largest difference in uptake was seen for P, Fe, Mn and S, where uptake in YPD was many fold higher in than in MSM. This finding suggests that *C. albicans* yeast operates different protein-synthesis and element storage strategies during growth on rich or poor media. In YPD, *C. albicans* reached a high cell density, generating 14 mg of dry weight biomass per ml of culture; in contrast, only 0.4 mg/ml was generated in MSM. YPD-grown cells may therefore assimilate higher levels of P, Fe, Mn and S to deal with the higher metabolic demand of growth in rich medium.

**Table 2:** Element concentration in rich (YPD) and poor (MSM) medium is not growth-limiting.

Element	YPD pH 5.6			MSM pH 4.5		
	Element in 100 ml YPD (μg) (molarity)	Element in stationary-phase yeast cell pellet from culture in 100 ml YPD (μg)	% available element taken up by cells	Element in 100 ml MSM pH 4.5 (μg) (molarity)	Element in stationary-phase yeast cell pellet from culture in 100 ml MSM (μg)	% available element taken up by cells
P	23,984 ± 1019, 7.7 mM	14,085 ± 305	59	6283 ± 247, 2 mM	317 ± 38	5
S	63,882 ± 4012, 20 mM	8676 ± 183	14	105,024 ± 17,584, 33 mM	271 ± 31	0.3
Mg	1953 ± 92, 804 μM	1426 ± 33	73	21 ± 6, 9 μM	16 ± 2	76
Ca	441 ± 19, 110 μM	31 ± 3	7	48 ± 4, 12 μM	1 ± 0.2	2
Fe	460 ± 32, 82 μM	343 ± 10	75	36 ± 4, 6.5 μM	2 ± 0.3	6
Cu	109 ± 14, 17 μM	20 ± 4	18	1 ± 0.6, 0.2 μM	0.3 ± 0.1	35
Zn	187 ± 6, 29 μM	126 ± 2	67	3 ± 0.7, 0.5 μM	2 ± 1	69
Mo	11 ± 0.6, 1.2 μM	0.1 ± 0.003	1	N.D.	N.D.	N.D.
Mn	5 ± 0.3, 0.9 μM	4 ± 1.8	79	0.3 ± 0.3, 0.05 μM	0.04 ± 0.007	16
Co	2 ± 0.1, 0.4 μM	0.04 ± 0.004	1	N.D.	N.D.	N.D.

The element composition of 100 ml fresh YPD (pH 5.6) or MSM (pH 4.5) medium, or of cell pellets of *C. albicans* grown to stationary phase in 100 ml of the same medium, were analysed by ICP-MS.

N.D = Not determined - below the limit of detection.

When the trace metals Fe, Zn and Mn were available at relatively high levels, 67–79 % of them were assimilated by the fungus (Table 2). During infection, these 3 ions are actively withheld by the human host (nutritional immunity). Because *C. albicans* has evolved as a commensal and opportunistic pathogen of humans, it is possible that this fungus has evolved to store these metals in excess for subsequent use in the nutrient-poor environment of the infected host.

### 3.3 Whole-cell trace element profiles and intracellular speciation in *C. albicans*, *C. neoformans* and *S. cerevisiae* grown as yeast

**Fig. 1:**
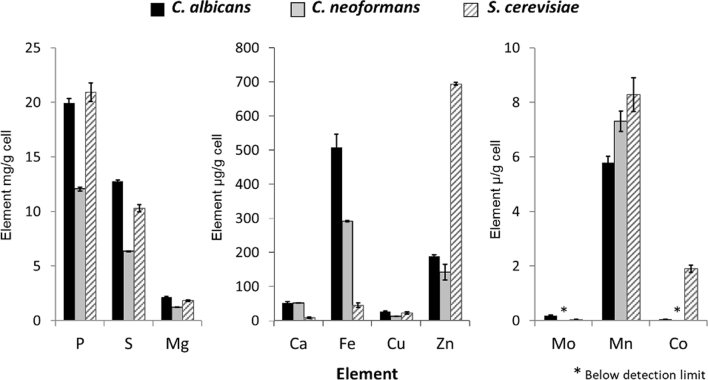
**Comparison of trace elements in yeast cells of *C. albicans, C. neoformans* and *S. cerevisiae*** Cells were grown in YPD pH 5.6 at 30 °C, pelleted and dried. Total elements were analysed by ICP-MS. Bars = SD, n = 3.

We next compared the trace element profile per gram of dried cell pellets for the two pathogenic yeasts, *C. albicans* and *C. neoformans,* and the model yeast, *S. cerevisiae,* grown in YPD at 30 °C (Fig. 1). *C. neoformans*, a basidiomycete, contained less of the most abundant elements, P, S and Mg, than the two ascomycetes, but there was little difference between the three species for Cu and Mo. In fact, the extremely low levels of Mo detected in these three yeast species may be due to the fact that most yeasts have lost the Mo cofactor biosynthetic machinery (Zhang and Gladyshev, 2008). The two pathogenic yeasts acquired significantly more Ca than the non-pathogenic *S. cerevisiae.* Interestingly, *S. cerevisiae* took up far more Zn than *C. albicans* or *C. neoformans*. This may be because *S. cerevisiae* tends to grow fermentatively, even in the presence of oxygen. Fermentation in yeast is mediated by Adh1 (alcohol dehydrogenase) – a zinc-dependent enzyme (Raj et al., 2014). Indeed, Adh1 levels in *S. cerevisiae* are estimated at 7.5 × 10^5^ protein molecules per cell, accounting for 1.5 × 10^6^ zinc ions (Eide, 2006). Therefore, *S. cerevisiae* may have assimilated high levels of Zn to fuel Adh1-mediated fermentation.

**Fig. 2:**
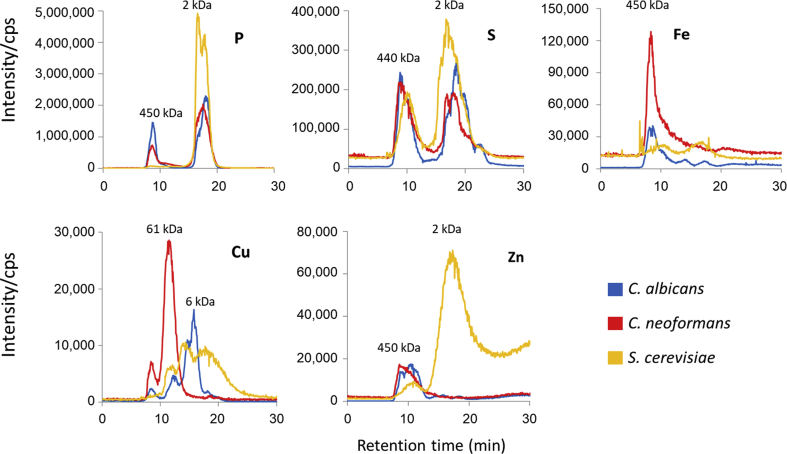
**Comparison of trace element speciation in the intracellular fraction of *C. albicans, C. neoformans and S. cerevisiae.*** Samples were analysed by HPLC coupled with an ICP-MS. A globular protein standard mix was used to determine molecular weight (indicated above chromatograms) of protein in size exclusion chromatography. Elution time of standards: Thyroglobulin (670 kDa) 8.5 min; y-globulin (158 kDa) 10.5 min; Vitamin B12 (1.35 kDa) 18.6 min. Sample-specific molecular weights are approximate and are affected by the globular or linear structures of molecules.

Of interest was that *C. albicans* assimilated more iron than the other two species. Of the three, *C. albicans* is the only species which has evolved as a commensal and opportunistic pathogen of humans and may store trace metals such as iron for subsequent use during infection. We next compared the abundance and sizes of the element-associated species for 5 trace elements in the intracellular fraction of the three yeasts (Fig. 2). Speciation analysis using ICP-MS coupled to a size-exclusion column can give basic information on the abundance and sizes, in the range of 500–0.5 kDa, of the molecules to which elements are bound. These divide into proteinaceous forms, such as metalloenzymes, metal transport/chaperone proteins and metal stress proteins, and non-proteinaceous molecules such as organic acids, phospholipids and polysaccharides. For S, the profiles of the three were similar, suggesting that the major intracellular binding-partners of these elements are consistent across all fungi, and possibly all eukaryotes. The major differences were in the distributions of P, Cu, Fe and Zn. For P, Fe and Zn, *C. albicans* and *C. neoformans* shared a similar species-size profile but *S. cerevisiae* was notably different. It lacked a high-molecular weight species for P and the major Fe-associated species that was seen in high abundance in *C. neoformans*, but instead showed a large, low-molecular weight peak for Zn. These results reflect the element distribution seen in the total cell analysis and indicate that the high amount of Fe in *C. neoformans* is bound to intracellular proteins that differ between the organisms. However, *S. cerevisiae* binds the majority of its Zn in the form of low-molecular weight compounds. The protein-bound Zn pattern was similar for *C. albicans* and *C. neoformans.* Cu was the only element to exhibit a different profile for each fungus, with multiple peaks of differing sizes in each. Thus, the complement of Cu-binding molecules was the most variable across the three fungi.

### 3.4 *C. albicans* hyphae assimilate a higher level of transition metals than *A. fumigatus*

**Fig. 3:**
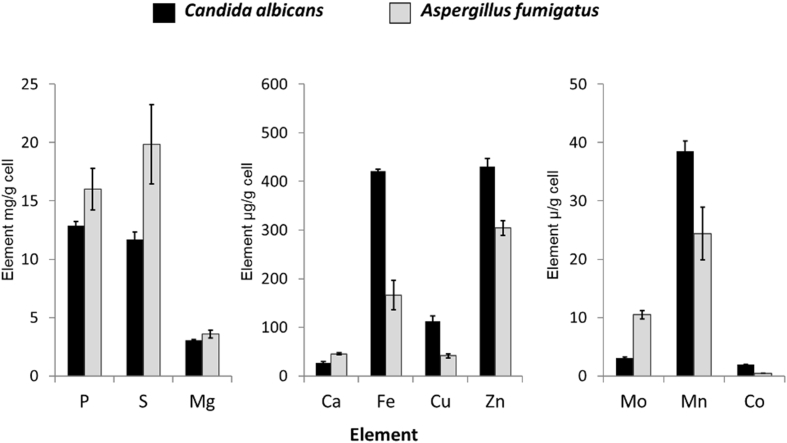
**Comparison of the trace element profile in hyphae of *C. albicans and A. fumigatus.*** Cells were grown in Complete Medium, pH 6.5 at 37 °C. Total elements were analysed by HPLC coupled with an ICP-MS. Bars = SD, n = 3.

We next compared the element profile of the hyphal filaments of *C. albicans* and the pathogen, *A. fumigatus*, grown in Complete Medium at 37 °C. *A. fumigatus* contained more of the non-metal elements, P and S, but the primary difference was in the transition metals, Fe, Cu, Zn and Mn, which were all at higher levels in *C. albicans* (Fig. 3). The exception was Mo, which was consistent with our previous findings where, in whole-cell analysis, *C. albicans* took up only 1 % of available Mo from rich media, and this element was not detectable in cells grown in MSM (Table 2). Most yeasts have lost the ability to utilise Mo (Zhang and Gladyshev, 2008) and we are not aware of any Mo-dependent processes in the yeast *C. albicans*. In contrast, Mo cofactor biosynthesis is important for the assimilation of nitrate and utilization of hypoxanthine as sole nitrogen sources in filamentous fungi (Probst et al., 2014).

### 3.5 Comparison of trace element profiles between *C. albicans* yeast and hyphae in a single growth medium

**Fig. 4:**
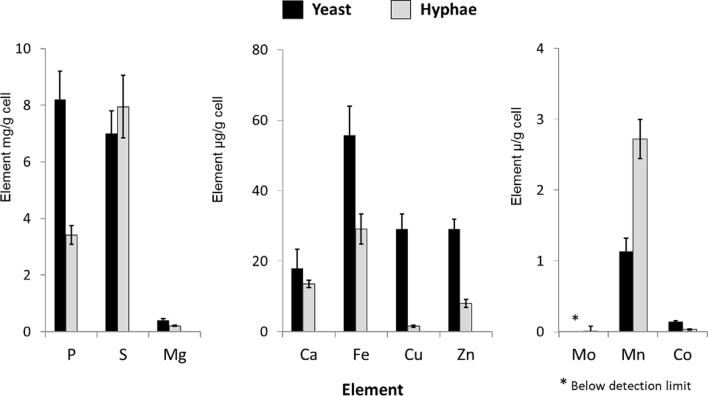
**Comparison of trace elements between yeast and hyphae morphology of *C. albicans***. Cells were grown in MSM as yeast (30 °C, pH 4.5) or hyphae (37 °C, pH 6.8). Total elements were analysed by HPLC coupled with an ICP-MS. Bars = SD, n = 3.

In *C. albicans*, the switch from yeast to hyphal growth morphology can be stimulated by a number of conditions, most of which involve a change in nutrient availability with a consequent induction of hypha-specific gene (HSG) expression (reviewed in Sudbery (2011)) (Martin et al., 2013, Sudbery, 2011). To compare assimilation by *C. albicans* yeast and hyphae, we quantified the element profile of cells grown in the same medium, Modified Soll’s Medium, where only a change in pH (from 4.5 to 6.8) and temperature (from 30 to 37 °C) was used to induce growth of yeast or hyphae, respectively (Fig. 4). Whilst pH can affect the solubility of some elements, the trace metals in MSM were all in the low micromolar range (Table 2), where pH should not significantly reduce bioavailability. Yeast cells contained higher levels of P, Fe, Cu, Zn and Co than hyphal cells when normalized to biomass. However, hyphae formed a three-fold higher biomass than yeasts in MSM (Table 1), yet yeast cells nevertheless assimilated 76 % and 69 % of Mg and Zn from the MSM medium (Table 2). Therefore, yeast and hyphal cultures assimilated similar total levels of these two metals.

We have previously analysed the molecular mechanisms of zinc uptake by *C. albicans* and found yeast cells grown in acidic media assimilate zinc almost exclusively via the Zrt2 transporter, whilst cells grown at neutral/alkaline pH express both the Zrt2 transporter and the Pra1-Zrt1 zincophore system (Crawford et al., 2018). Therefore, due to the media pH used to generate the two morphologies in this study, zinc was likely assimilated by yeast via Zrt2 and by hyphae via the Pr1-Zrt1 zincophore and by Zrt2.

In contrast to the finding for zinc, Mn was present at double the amount in hyphae compared to yeast. Combined with the fact that hyphae formed a three-fold higher biomass than yeast means that hyphae took up significantly more Mn from the culture. Although Mn import by *C. albicans* has not been studied directly, this fungus has an orthologue of the *S. cerevisiae* Smf1 manganese importer (called Smf12 in *C. albicans*) which it may use for assimilation of this metal. Interestingly, *SMF12* is upregulated in alkaline conditions in *C. albicans* (Bensen et al., 2004, Supek et al., 1996), which is in agreement with our observation that neutral/alkaline pH-induced hyphae took up more Mn. Once inside the cell, manganese may be shuttled to the vacuole via Ccc1 or into the Golgi via the P-type Mn–Ca transporter Pmr1. In the Golgi, Mn acts as an important cofactor for mannosyltransferases involved in cell wall protein glycosylation (Bates et al., 2005). Two other Mn-dependent proteins have been characterized in *C. albicans*. Sod2 and Sod3 are super-oxide dismutases that localize to the mitochondria and cytoplasm, respectively. Alkaline conditions down-regulate *SOD2* but *SOD3* is up-regulated in stationary-phase (Lamarre et al., 2001). The regulation and localization of these proteins suggest that the cellular role of Mn differs between hyphae and yeast but further study is required to define this more clearly.

### 3.6 Trace element profiles in response to excess calcium and cell wall stress conditions in *C. albicans*

**Fig. 5:**
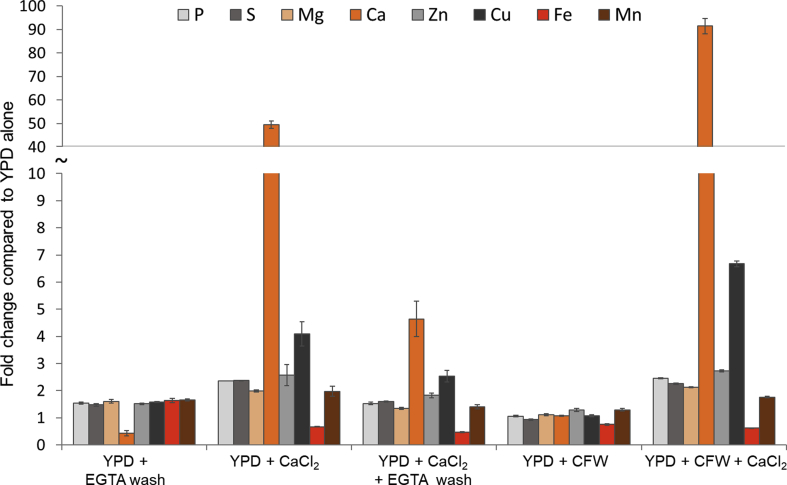
**The effect of stress on trace element composition in *C. albicans* yeast cells. *C. albicans*** was grown as yeast in YPD at 30 °C. Cell stress was induced by 100 μg/ml CFW and/or 0.2 M CaCl_2_. Where indicated, cells were washed with 20 mM EGTA after stress treatment but before drying. Compositional change is shown as the fold change normalised to cells grown in YPD only. Bars = SD, n = 3.

Ca signalling has been implicated in a number of cell stress responses, through its binding to calmodulin to activate the phosphatase, calcineurin (Blankenship and Heitman, 2005, Cruz et al., 2002, Kretsinger and Nockolds, 1973, Munro et al., 2007, Sanglard et al., 2003). In conjunction with the cell wall-binding compound, Calcofluor White (CFW), the presence of high extracellular Ca increases levels of chitin in the *C. albicans* cell wall, conferring strength and hence resistance to the echinochandin class of antifungal drugs (Lee et al., 2012). We therefore investigated the role of Ca and cell wall stress on *C. albicans* elemental profile. We first tested the effect of Ca. Cells were cultured in YPD, washed twice in EGTA (a commonly used calcium chelator, but which may chelate other cations) and their elemental profile compared to YPD alone. EGTA caused a moderate but consistent (∼1.5 fold) increase in the levels of all elements, with the exception of calcium, which decreased by over 50 % (Fig. 5). This is interesting because EGTA is a cell impermeable chelator. Therefore, our observation that EGTA “stripped” the cell of calcium indicates that either (i) its extracellular chelating activity triggered calcium efflux, or (ii) a significant fraction of cell-associated calcium is present in the fungal cell wall. Either possibility is plausible: for example, addition of the extracellular Zn chelator, EDTA, to *C. albicans* cells triggers rapid (<1 min) changes in intracellular Zn pools (Kjellerup et al., 2018). However, we think it more likely that a portion of Ca was bound to the cell wall and accessible to EGTA chelation.

We then tested the effect of exogenous Ca in the culture medium. Unsurprisingly, cell-associated Ca levels increased (49-fold). We also observed a two-fold increase in cellular P, S, Mg, Zn and Mn, a 4-fold increase in Cu and a 33 % reduction in Fe. Next, we cultured the cells in Ca-rich medium, as before, but then washed the cells with EGTA. The EGTA wash did not have a large impact on non-Ca elements, except for Cu, which decreased compared to YPD + Ca alone. Strikingly, the EGTA removed ∼90 % of cell-associated Ca. Although we cannot rule out extracellular EGTA triggering rapid efflux of intracellular Ca, these data suggest that, when grown under high Ca, *C. albicans* stores, or adsorbs, high levels of the cation in an extracellular, EGTA-labile location, most likely the cell wall. Although cations are known to bind to cell wall components, such as phosphomannan, we are not aware of reports of a unicellular fungus storing 90 % of their “cellular” Ca extracellularly.

We next assessed the effect of cell wall stress. Cells cultured in the presence of the cell wall stressor, CFW, did not exhibit appreciable changes in their elemental profile. Simultaneous CFW + Ca stress did not affect the profile of most elements compared to Ca-treatment alone. Cu was again the exception, as it increased further to 6.7-fold above the YPD only control. Interestingly, combined CFW + Ca resulted in a 91-fold increase in cellular Ca. This value is almost double that of Ca-treatment alone. The observation that raised Ca positively correlated with an increase in Cu (r = 0.98) suggested a direct link between these elements. Activation of calmodulin and/or calcineurin pathways may have affected the expression of iron and copper transporter genes. For Fe, both CFW and high extracellular Ca resulted in decreases of 33–53 %, suggesting the effects of these treatments are indirect. For example, perturbation of the cell wall in general may be responsible for Fe loss on treatment with CFW. For treatment with high Ca, these cations at the cell surface may have hindered the diffusion of charged Fe ions across the cell wall, limiting uptake (cation-cation repulsion), or have stoichiometrically blocked access to iron permeases. Stress is known to impact cell wall architecture via a number of mechanisms and Ca + CFW increases chitin content in the cell wall (Lee et al., 2012, Roncero, 1984). Additionally, iron limitation (which occurs in the presence of high Ca, Fig. 5) increases cell wall thickness (Pradhan et al., 2019). Therefore, a simple thickening of the cell wall may account for increased extracellular Ca storage.

In summary, combined CFW + Ca stress resulted in very high levels of Ca, which were likely associated with the cell wall, and a concomitant rise in Cu and fall in Fe. However, at this stage the mechanism underlying the relationship between Ca, Cu and Fe levels remains enigmatic.

### 3.7 Role of the fungal cell wall in calcium homeostasis

Our data strongly suggest that, depending on the environmental conditions, *C. albicans* can store high levels of Ca in an extracellular, EGTA-accessible structure. The fungal cell wall is, to our knowledge, the only extracellular organelle that might be predicted to serve this function.

Fungal cells walls are comprised primarily of polysaccharides bearing the negatively charged hydroxyl, carbonate and phosphate groups that, at neutral pH, are strongly attractive to the hard metals, Na, Mg and Ca (Bartnicki-Garcia, 1970). In gram-positive bacteria, Mg contributes to cell wall stability and Mg and Ca bind the structural peptidoglycans to the outer cell membrane (Thomas and Rice, 2014). In fungi, the other primary cell wall components are chitin, proteins and, in some fungi, melanin (Gow et al., 2017, Bhattacharjee et al., 1984). Chitin is regarded as crystalline and therefore less reactive than glycan polymers and proteins, which contain the hydroxyl, amine and sulphur groups that attract the transition metals. However, the extent of metal binding depends not only on the presence of these functional groups but on the spatial organisation and density of the cell wall, properties that are key mediators of its ion-exchange capacity (Remacle, 1990). Our data adds to the body of evidence identifying cell walls as storage organelles for essential cationic trace elements. Whether or not the *C. albicans* cell wall is specifically regulated to facilitate the capture of essential trace elements in different host niche environments is not known. However, in *A. fumigatus*, melanin, a specific cell-wall component not found in *C. albicans*, sequesters Ca within the host phagosome to prevent Ca-calmodulin activation of a specialized phagocytic pathway (Kyrmizi et al., 2018). In addition, plant cells have been reported to remodel their cell walls to act as a sink for toxic heavy metals (Krzesłowska, 2011). Given the essentiality of acquiring trace elements, it seems likely that fungal cells optimally organise their cell wall to assist with trace element acquisition and storage.

Trace elements associated with the cell wall may also play an active role as signalling molecules if released by perturbation of the cell wall, for example by tip contact with an external obstacle (Thomson et al., 2015 Brand et al., 2007) or by an environmental change in ambient pH. The structural properties of cell walls vary enormously within and between fungal species in response to growth conditions. In *C. albicans*, growth in YPD containing 2 % glucose produces a 100 nm thick, spongy cell wall but growth in lactate reduces wall thickness to 50 nm and increases the Young’s modulus (Ene et al., 2012). The specifics of any role for the cell wall in trace element storage and/or signalling is therefore likely to vary enormously by fungal species and their growth response to specific environmental conditions.

In summary, our study has described the degree of trace element assimilation from commonly used laboratory media by three important human fungal pathogens. Because cellular trace metal levels can significantly alter fungal stress responses and pathogenicity, it is important for researchers to consider how pre-culture in laboratory media affects the elemental profile of cells in the context of the experiment at hand. We also show that the Ca chelator EGTA can remove over 90 % of a cell population’s Ca content, suggesting that *C. albicans* can store large amounts of Ca outside the cell and likely in the cell wall. Some body sites can be relatively rich in calcium and the implications of the cell wall acting as an extracellular metal storage, and possibly release, compartment will be intriguing to dissect in the future.
